# Magnetoelectric Response of Laminated Cantilevers Comprising a Magnetoactive Elastomer and a Piezoelectric Polymer, in Pulsed Uniform Magnetic Fields

**DOI:** 10.3390/s21196390

**Published:** 2021-09-24

**Authors:** Gašper Glavan, Inna A. Belyaeva, Kevin Ruwisch, Joachim Wollschläger, Mikhail Shamonin

**Affiliations:** 1 East Bavarian Centre for Intelligent Materials (EBACIM), Ostbayerische Technische Hochschule (OTH) Regensburg, Seybothstr. 2, D-93053 Regensburg, Germany; inna.belyaeva@oth-regensburg.de; 2 Fachbereich Physik der Universität Osnabrück, Barbarastr. 7, D-49076 Osnabrück, Germany; kruwisch@uos.de (K.R.); jwollsch@uni-osnabrueck.de (J.W.)

**Keywords:** magnetoactive elastomer, piezoelectric polymer, laminated structure, cantilever, direct magnetoelectric effect, magnetic field sensor

## Abstract

The voltage response to pulsed uniform magnetic fields and the accompanying bending deformations of laminated cantilever structures are investigated experimentally in detail. The structures comprise a magnetoactive elastomer (MAE) slab and a commercially available piezoelectric polymer multilayer. The magnetic field is applied vertically and the laminated structures are customarily fixed in the horizontal plane or, alternatively, slightly tilted upwards or downwards. Six different MAE compositions incorporating three concentrations of carbonyl iron particles (70 wt%, 75 wt% and 80 wt%) and two elastomer matrices of different stiffness are used. The dependences of the generated voltage and the cantilever’s deflection on the composition of the MAE layer and its thickness are obtained. The appearance of the voltage between the electrodes of a piezoelectric material upon application of a magnetic field is considered as a manifestation of the direct magnetoelectric (ME) effect in a composite laminated structure. The ME voltage response increases with the increasing total quantity of the soft-magnetic filler in the MAE layer. The relationship between the generated voltage and the cantilever’s deflection is established. The highest observed peak voltage around 5.5 V is about 8.5-fold higher than previously reported values. The quasi-static ME voltage coefficient for this type of ME heterostructures is about 50 V/A in the magnetic field of ≈100 kA/m, obtained for the first time. The results could be useful for the development of magnetic field sensors and energy harvesting devices relying on these novel polymer composites.

## 1. Introduction

Multiferroic magnetoelectric (ME) composites attract large attention both from academe and industry due to their applications in sensor technology, high-frequency engineering, energy harvesting devices, random access memories, etc. [1,2,3,4,5,6]. Conventional ME layered composites are built from rigid constitutive materials (Young’s modulus *Y* ~ 10^11^ Pa). A significant reduction of the effective Young’s modulus below 10^9^ Pa would allow one to decrease the resonance frequency of a composite layered multiferroic structure (~1–100 kHz in conventional multilayered composites), where the efficiency of ME coupling is maximum, to the low-frequency range of 1 to 100 Hz. This would be advantageous for a number of applications such as vibration energy harvesting [7] or low-frequency magnetic-field sensing [8,9]. This justifies the interest in mechanically soft ME materials because of their absence in nature. At the present state of technology, polymer materials would be a natural choice. A comprehensive review of piezoelectric (PE) polymer materials is given in Ref. [10]. Low resonance frequencies (in order of a few hundred Hz) can also be achieved in compositions of a PE polymer with a ferromagnetic metal if the thickness of the magnetostrictive (MS) layer is of the order of several micrometers [11].

Two papers considered theoretically novel concepts on how to design ME soft materials. Alameh et al. [12] proposed an approach that does not require the materials themselves to be ME, or piezoelectric (PE) or exhibit any exotic atomistic features that conventional hard crystalline multiferroics do. As long as the magnetic permeability of the soft matter is larger than that of vacuum, an emergent ME effect should appear due to the interaction of deformation and a pre-existing electric field. Rambausek and Keip [13] demonstrated that the strain-mediated ME coupling induced by finite deformations in soft composites could be of significant magnitude due to the shape effect as a specific non-local phenomenon in magneto- and electro-elasticity. It was found that, among ellipsoidal bodies, the shape-influenced ME coupling should be most pronounced for those of spherical to moderately prolate shape.

Zhang et al. proposed an alternative way to design compliant ME composites and demonstrated several experimental realizations [14,15,16]. The idea was to embed a hard-magnetic material (NdFeB), surrounded by an electrically conductive helix coil, into a soft silicone matrix. When the resulting material was deformed, e.g., compressed along the coil axis, an open-circuit output voltage up to ≈100 µV or the short-circuit output current up to ≈60 µA was observed between coil terminals due to electromagnetic induction. Therefore, the authors observed a direct PE effect and not a ME effect in their material realizations. It is plausible that such materials may display ME properties due to the interaction of hard-magnetic filler material with an external magnetic field.

A new, alternative line of research explores the so-called magnetoactive elastomers (MAEs), in which micrometer-sized ferromagnetic particles are embedded into a soft polymer matrix [17,18,19,20,21,22,23]. MAEs are known to possess superior properties as far as the magnitude of the magneto-mechanical effects is concerned. For example, giant (~10^−1^) magnetostriction can be observed using compliant MAEs [24]. Relevant results were reported by Sheridan et al. [25] and Kalita et al. [26], who studied the significant bending of MAE cantilevers in external magnetic fields. To the best of our knowledge, the ME coupling in a layered composite with an MAE layer was first reported by Feng et al. [27] who cured an MAE layer on a polyvinylidene fluoride (PVDF) film. Makarova et al. published a series of papers [28,29,30] devoted to the development of ME polymer-based composite materials using silicone matrices. Initially, they reported multiferroic ferroelectric/ferromagnetic-polymer composite systems, consisting of ferroelectric lead zirconate titanate (PZT) particles, ferromagnetic NdFeB (or barium ferrite) particles, and silicone matrix [28]. In the second paper, the authors fabricated composite materials based on ferroelectric porous structure and either MAE or magnetic-fluid filler [29]. The maximum value of the coefficient of the converse ME transformation for the sample with Fe microparticles was found to be ≈36 pT·m/V, which is three orders of magnitude smaller than can be observed in conventional (stiff) laminated composites [31]. Very recently, the same group reported the first realization of layered structures comprising a commercially available PE polymer (PVDF) and MAE and pointed out their perspectives for sensors and energy harvesting devices [30]. The direct ME effect was observed as a voltage appearing between the electrodes of the PE polymer due to the bending of the composite structure in a non-uniform magnetic field. Unfortunately, the magnetic field gradient was not specified. The amplitude of voltage oscillations during magnetic-field pulse excitations (pulse duration ~10 ms) reached about 650 mV. The work [30] demonstrated that a significant direct ME effect is feasible in multiferroic composite materials comprising MAE layers. However, further research is required, because the unconventional experimental configuration in [30] (in particular, the external magnetic field is non-uniform) did not allow one to determine the ME coupling coefficient. Additionally, it remains unclear how the physical properties of the MAE layer(s) should be optimized in order to achieve the maximum ME voltage coefficient.

Very recently, Tan et al. [32] published a new concept for developing soft ME materials, which they called the magnetoelectric electret (MEE). The key idea was to deposit net charges on the interface between two layers of materials, which are different in magnetic properties [12]. One layer was a polytetrafluoroethylene (PTFE) thin film and the other layer was an MAE. Before bonding these two layers together, a layer of surface charge was deposited onto one surface of the PTFE thin film by the corona charging technique. In a uniform magnetic field, applied perpendicularly to the charged surface, the two-layered structure deformed asymmetrically, i.e., one layer (MAE) deformed more than the other layer (PTFE). This magnetic-field-induced asymmetric deformation resulted in voltage differences between the upper and lower surfaces of the MEE. The theoretical explanation was based on the concept of a Maxwell stress [33]. The material exhibited a room temperature ME coefficient of about 0.24 V/A at the magnetic field of ≈48 kA/m and a low frequency of ≈1 Hz.

Hitherto, the usage of MAEs in mechanically soft ME multiferroic composites is in the initial stage, although the published results are very promising. The aim of this paper is to investigate in detail the behavior of laminated structures comprising an isotropic MAE layer and a commercially available PVDF-based vibration sensor in pulsed uniform magnetic fields, directed almost perpendicularly to the sample plane. In particular, the maximum induced voltage exceeds that reported in [30] by more than eight-fold. Moreover, we report the first, to the best of our knowledge, measurement of the magnetically induced ME coupling coefficient in such a material [34] and its peculiar dependence on the external magnetic field. The paper is organized as follows: In the following section, the fabrication methods of composite cantilevers and the experimental setup are described. In particular, the effect of the fabrication method on the ME response is addressed. In Section 3, the magnetic properties of employed MAE materials are presented. Dependences of the induced voltage and the cantilever deflection on the composition of the MAE layer (volume fraction of the filler and the softness of the elastomer matrix) as well as on its thickness are presented. From these dependencies, the guidelines for designing MAE-PVDF structures with the maximum response to the (almost) perpendicular magnetic field are derived. Transient behavior is studied. In Section 4, the underlying physical effects of the cantilever’s deflection and generated voltage are discussed. Furthermore, we present the first measurement of the ME coupling coefficient for this novel type of structure and discuss its physical significance. Conclusions are drawn in Section 5, where also the future work is discussed.

## 2. Materials and Methods

### 2.1. Fabrication of Composite Structures

Planar structures comprised an MAE layer bonded to a commercially available PVDF-based vibration sensor (LDT0-028K, Measurement Specialties, Hampton, VA, USA) [35]. The latter is a flexible component comprising a 28 μm thick PE PVDF polymer film with screen-printed silver ink electrodes, laminated to a 0.125 mm polyester substrate, and fitted with two crimped contacts. The PE film is displaced from the mechanical neutral axis, therefore bending creates very high strain within the PE polymer and high voltages are generated. In the following, this electronic component will be denoted as the PE polymer (PEP).

**Figure 1:**
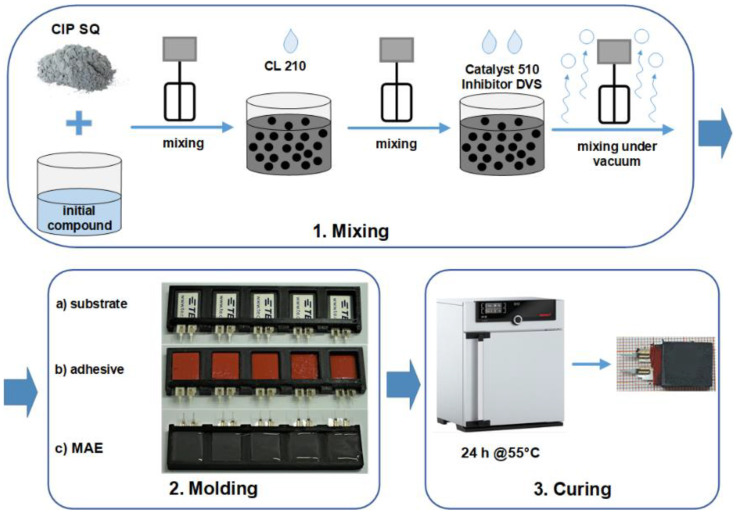
Technological process of the fabrication of MAE-PEP layered structures.

The synthesis of MAE materials followed the known path [36,37]. The entire technological process is illustrated in Figure 1. The base polymer VS 100000 (vinyl-functional polydimethylsiloxane) for addition-curing silicones, the chain extenders Modifier 715 (SiH-terminated polydimethylsiloxane), the reactive diluent polymer MV 2000 (monovinyl functional polydimethylsiloxane), the crosslinker CL 210 (dimethylsiloxane-methyl hydrogen siloxane copolymer), the Pt catalyst 510, and the inhibitor DVS were provided by Evonik Hanse GmbH, Geesthacht, Germany. The silicone oil AK 10 (linear, nonreactive polydimethylsiloxane) was purchased from Wacker Chemie AG, Burghausen, Germany. A carbonyl iron powder (CIP) (type SQ, mean particle size of 3.9–5.0 μm, BASF SE Carbonyl Iron Powder and Metal Systems, Ludwigshafen, Germany) was used as the ferromagnetic filling. CIP is fabricated by thermal decomposition of iron pentacarbonyl (Fe(CO)_5_), which is previously distilled to high purity [38]. The minimum iron content in SQ particles is 99.5% [39]. It is well known that CIP particles have a spherical shape, which was obtained by scanning electron microscopy [40,41].

The polymer VS 100000, the polymer MV 2000, the modifier 715 and the silicone oil AK 10 were put together and blended with an electric mixer to form an initial compound. In the next step, the initial compound was mixed together with the CIP particles and the CL 210. The crosslinking reaction was activated by the Pt-Catalyst 510. For the control of the Pt-catalyst’s activity, the inhibitor DVS was used. PE substrates were fixed in a specifically designed plastic mold and their top side was covered with a thin layer of a silicone adhesive (Permatex^®^ High Temp Red RTV Silicone Gasket Maker, Solon, OH, USA). The uncured MAE in form of a viscous liquid was poured onto the adhesive layer to the desired thickness of the MAE layer. The rest of the material was put into another mold (Petri dish with a diameter of about 33 mm, Greiner Bio-One International GmbH, Kremsmünster, Austria) for further characterization and quality control. The resulting structure was cured in a universal oven (Memmert UF30, Memmert GmbH, Schwabach, Germany) for 24 h at 55 °C with air circulation. The curing was performed in the absence of a magnetic field. Therefore, the MAE material can be considered as random heterogeneous, i.e., no chain-like aggregates could be formed during crosslinking.

For the purposes of this study, two types of matrix were synthesized. We denote them as “soft” and “rigid” to emphasize the difference in the elastic moduli of the matrices. The word “soft” refers to the samples with the lower elastic modulus, while the word “rigid” refers to the samples with the higher elastic modulus. The variation in the elastic modulus was achieved by changing the amount of cross-linker added to the initial compound.

Table 1 summarizes typical mechanical properties of the two matrices and the MAE materials on their basis. The shear storage modulus $G_{0}^{\prime }$ was obtained from rheological measurements as described in Section 2.2 below.

As expected, the effective shear modulus of the MAE material increases with increasing concentration of iron particles and, for the same fraction of the filler, it is higher for composites with the rigid matrix.

Note that the curing of the MAE layer can be carried out directly on the PEP, i.e., step (2b) can be skipped. However, as it will be explained below, a thin layer of a silicone glue improves adhesion between MAE and PEP layers. Consequently, a thin layer of a silicone glue allows one to enhance the ME response of the composite cantilever.

In the present paper, the results obtained on 19 different cantilevers from 6 different material combinations are presented.

### 2.2. Material Characterization

To characterize the MAE materials using rheological measurements, fully cured MAE samples in the form of disk-shaped plate with a diameter of 20 mm and a thickness of 1 mm were cut from a Petri dish. Magnetorheological measurements were performed using a commercially available rheometer (Anton Paar, model Physica MCR 301). The angular oscillation frequency ω was kept constant at 10 rad/s. To avoid slippage, the normal force of approximately 1 N was applied. The moduli were measured at constant strain amplitude γ = 0.01%, which corresponds to the linear viscoelastic regime. The measurement time was 20 s per data point.

The MAE samples for magnetic measurements were also cut out of a mold. They were of cylindrical shape with a diameter of approximately 6 mm and a height of about 1 mm. Magnetization curves were measured in-plane (perpendicular to the cylinder axis) at room temperature by varying the external magnetic field $\mu_{0}H$ from 1.5 T to −1.5 T and back again, using a vibrating sample magnetometer (model 7407, Lake Shore Cryotronics, Inc., Westerville, OH, USA).

### 2.3. Experimental Setup

**Figure 2:**
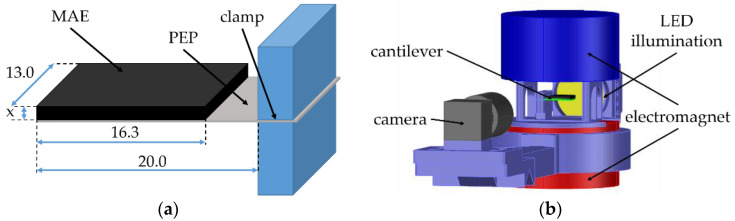
(**a**) Sketch of the resulting MAE-PEP cantilevers. All dimensions are given in mm. The thickness x of the MAE layer is variable. (**b**) Scheme of the experimental setup.

To realize a cantilever, a fabricated structure was clamped at one end (on the side of electrical connectors) in a specifically designed 3D printed holder from the polylactic acid (PLA) material. The resulting dimensions of the cantilever are shown in Figure 2a. The MAE material was always bonded to the “negative” side of the PE element. The side “polarity” is defined by the PE element manufacturer. On this side, the manufacturer’s logotype is imprinted, see Figure 1. The cantilever was always clamped in such a way that the MAE was on its top side.

The cantilever in the holder was placed between the poles of a large electromagnet (EM2 model, MAGMESS Magnetmesstechnik Jürgen Ballanyi e.K., Bochum, Germany), cf. Figure 2b. The pole diameter was 9.2 cm and the vertical distance between the poles was 3.4 cm. The vertical magnetic field of the electromagnet was highly uniform over the entire space where the cantilever could move. We verified that the uniformity of a magnetic field was better than 99.9% over the right circular cylinder with a radius of 2 cm and a height of 3.4 cm, which symmetry axis coincided with the symmetry axes of the magnetic poles. The electromagnet was powered by a bipolar power supply (FAST-PS 1k5, CAEN ELS s.r.l., Basovizza, Italy). The deflection of a cantilever was recorded using a CMOS-based camera (Alvium 1800 U-319 m, Allied Vision Technologies GmbH, Stadtroda, Germany) with a suitable lens (Edmund Optic Double Gauss Focusable, 25 mm C-mount F4.0 1.3”, Barrington, New Jersey, NJ, USA) and a backlight LED illumination (LED Illuminant G4 Pen). The generated voltage by a PE element was measured using a data acquisition board (NI USB-6212, National Instruments, Austin, TX, USA). The generated electrical charge was obtained by electrical current integration using a charge amplifier (Kistler 5018A, Winterthur, Switzerland). The entire experimental process was automated using the LabVIEW software.

The “negative” side of the PE element was connected electrically to the conducting shield (“ground”) of the coaxial cable, while the “positive” side was connected to the core electrode (“signal”). With such a choice of the signal polarity, the first voltage peak was positive, when the composite cantilever was deflected down upon application of an external magnetic field. When an external magnetic field was switched off then, the second voltage peak was negative, see Section 3.1 for details. If the cantilever was deflected up when a magnetic field was switched on and off, the polarity of two voltage peaks was changed to the opposite.

### 2.4. Image Processing

**Figure 3:**
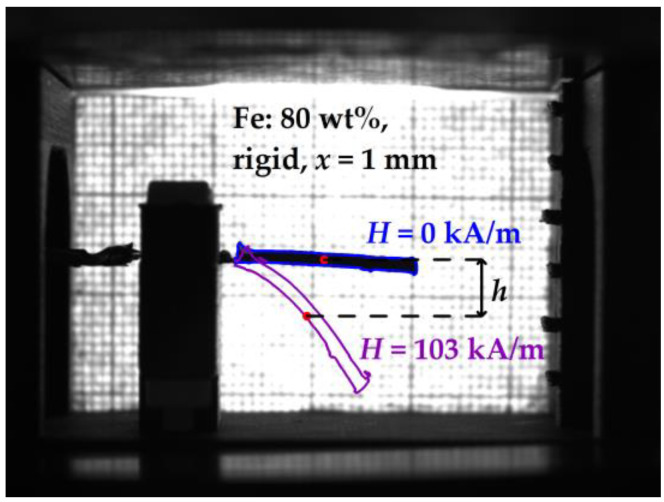
Illustration of the image processing for determining the deflection of the “mass center” h of the cantilever. *H* denotes the magnetic field strength.

To quantify the deflection of the composite cantilever, image processing was performed. For this purpose, a Python script using the OpenCV library was written. The cantilever’s deflection h was determined as the vertical displacement of the “mass center” of the cantilever from its initial position in the absence of a magnetic field as presented in Figure 3. The “mass center” shown as a colored dot in Figure 3 was defined as the average pixel coordinate of the cantilever’s contour. We found that such an image analysis works more reliably than tracking the tip of the cantilever. The deflection of the free tip of the cantilever is approximately twice larger than the deflection of the “mass center”.

### 2.5. Measurement Protocol

**Figure 4:**
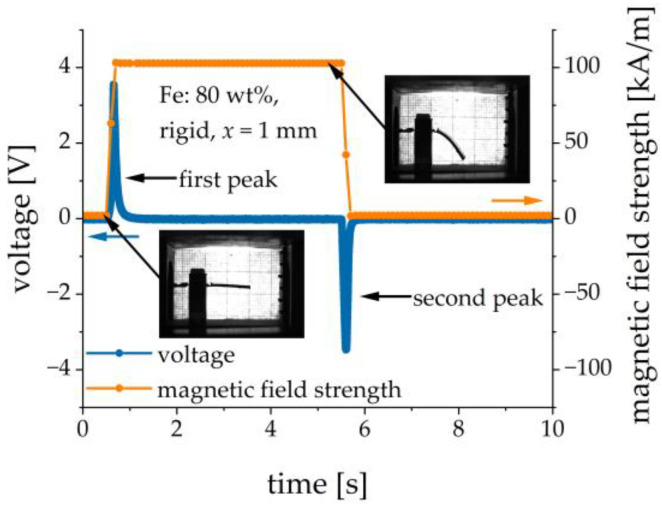
Example of transient behavior of the applied magnetic field and the resulting voltage response.

The experimental setup was calibrated in such a way that the electric current through the electromagnet coils could be re-calculated into the magnetic field strength. The resulting magnetic field strength was directly proportional to the excitation current. Pulsed magnetic field excitations were studied. The magnetic field was first switched on. In general, the rate of the electrical current change could be set to some value. In the following experiments, the electrical current ramp rate was set to the highest possible value of 15 A/s, which corresponded to the magnetic-field ramp rate of approximately 770 ${\mathrm{kAm}}^{-1}s^{-1}$. The resulting ramp of the magnetic field was practically linear. The magnetic field was kept constant for a few seconds and then it was switched off at the same rate. Normally, the magnetic field remained switched on for 2 s and the switch-off pauses between different settings of the magnetic field were also 2 s. Only in Section 3.5, where the transient behavior was studied, the magnetic field remained switched on for 5 s, in order to determine the time constant more precisely. The duration of the transient process for the electrical current (~0.1 s) was much less than a few seconds. The excitation current, the cantilever’s deflection (via image analysis) and the output voltage were measured as functions of time. Figure 4 shows the typical transient behavior of the applied magnetic field and the resulting output voltage. Two voltage peaks of different polarities were observed, see Figure 4. In the following, we refer to the measurements of the amplitude of the first voltage peak and to the maximum deflection of the cantilever in a particular magnetic field, which was normally reached just before the magnetic field was switched off. To remove some parasitic noise at mains frequency, a notch peak filtering at 50 Hz with the 3dB bandwidth of 10 Hz was employed for the output voltage.

Contrary to the results reported in [30], we did not observe pronounced damped oscillations of the output voltage at a frequency of about 40 Hz, both in unfiltered and filtered signals, when the magnetic field was switched off. In most cases, we just observed the monotonic decay of the trailing edges of output voltage pulses. For some samples with heavier MAE layers (i.e., with the large MAE thickness and higher concentration of Fe particles), we detected oscillations of the output voltage at roughly 25 Hz overlapping the decay of the trailing edge. We attributed these oscillations to those at the resonant frequency of the composite cantilever with added mass, as is described in the datasheet for this PE element. These oscillations at the resonant frequency seem to be strongly damped by the presence of a relatively thick viscoelastic MAE layer. However, a possible presence of the damped oscillations of the composite cantilever does not influence the following considerations or conclusions.

## 3. Results

### 3.1. Magnetic Properties of MAE Materials

**Figure 5:**
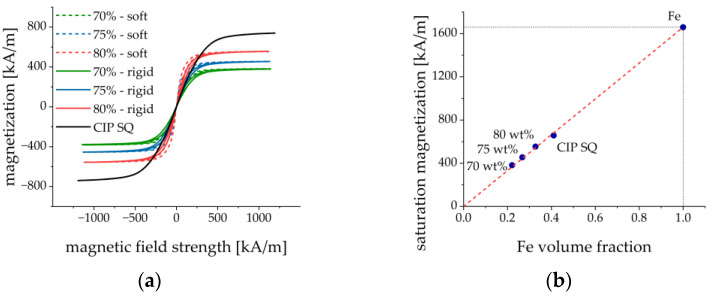
(**a**) Magnetization loops of fabricated MAE materials and carbonyl iron powder. The magnetization curves of MAE are plotted versus the internal magnetic field, while the magnetization curve for CIP is presented versus the external magnetic field. (**b**) Dependence of the saturation magnetization on the volume fraction of iron particles. The dashed line represents the linear regression from which the saturation magnetization of CIP particles was calculated (point Fe).

The magnetic properties of the six developed MAE materials were investigated. The results are shown in Figure 5. A typical “pinched” hysteresis loop behavior was observed as reported and explained in [42] by the restructuring of filler particles in external magnetic fields and different scenarios of formation and destruction of elongated particle aggregates in ascending and descending magnetic fields, respectively. The magnetization curves for different MAEs in Figure 5a are shown versus the internal magnetic field $H_{int}=H_{ext}-N_{∥}M$, where the in-plane demagnetizing factor $N_{∥}$ perpendicular to the cylinder axis was estimated to be about 0.137 using a simplified formula by Sato and Ishii [43]. The estimated value of the demagnetizing factor is quite close to the values obtained in the classical work by Chen et al. [44]. It is a reasonable assumption because the maximum effective magnetic permeability of the materials is not too large. The magnetization curve for the CIP is shown against the external magnetic field because the shape of the powder holder is rather complicated and the demagnetizing factor cannot be estimated. The curve for CIP is included firstly to demonstrate the negligible hysteresis loop as compared to MAEs and secondly to estimate the saturation magnetization $M_{s}$. The coercive force $H_{c}$ of CIP was ≈0.5 kA/m and the remanence $B_{r}$ was ≈2 mT. The coercive force and the remanence of the MAEs were of the same order of magnitude. The remanent magnetization of CIP was less than 0.23% of the saturation magnetization.

**Figure 6:**
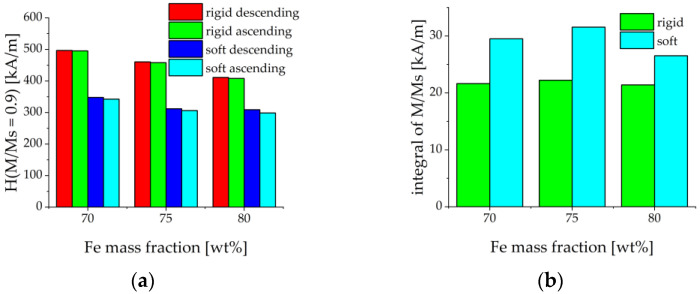
(**a**) Values of the internal magnetic field strength when magnetization reaches 90% of saturated magnetization for ascending and descending branches of the hysteresis loop. (**b**) Area of the normalized magnetization loop in the first quadrant.

Figure 5b depicts the dependence of the saturation magnetization on the concentration of iron particles. $M_{s}$ was determined as the magnetization in the maximum external magnetic field of 1200 kA/m. Figure 5b demonstrates that the saturation magnetization is indeed proportional to the volume fraction of iron particles, as it should be. The saturation magnetization of carbonyl iron particles was determined to be 1659±25 kA/m ($\mu_{0}M_{s}\approx 2.08$ T) which agreed well with the literature [45]. It could be expected that the magnetically induced restructuring of ferromagnetic particles should be pronounced stronger in MAEs with a softer polymer matrix. This was indeed observed in the results of Figure 5. The evaluation of the magnetization loops showed that MAEs with the soft matrix approached the magnetic saturation earlier than MAEs with the rigid matrix. The magnetic fields, where 90% of the saturation magnetization was achieved, are roughly 50% lower than for MAEs with soft matrix and the same volume fraction of magnetic particles, see Figure 6a. Further, each magnetization curve had been normalized on its saturation magnetization. Then the loop area in the first quadrant was calculated by the following method. The results between the measured points were first interpolated using splines with a step of 10 kA/m for both descending and ascending magnetic field branches. Then the trapezoid rule was used to calculate the integral numerically. It turned out that the loops for the MAEs with a soft matrix are roughly 40% broader (i.e., their area is larger) than the loops for the MAEs with a rigid matrix, see Figure 6b. This can be explained by higher losses in the softer viscoelastic matrix due to the larger displacements of magnetized particles as compared to the more rigid matrix. We conclude that the mechanical properties of the elastomer matrix and the volume fraction of the iron particles are visible in the magnetization curves.

### 3.2. Optimization of Fabrication Method

**Figure 7:**
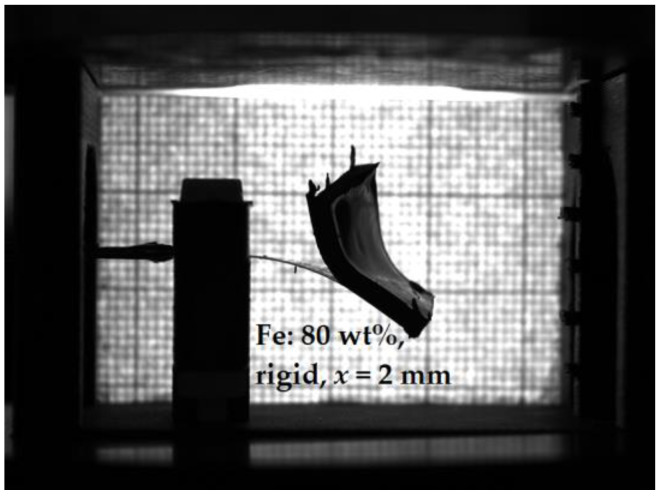
Example of poor adhesion between an MAE layer and a PE substrate after several loading cycles. Parameters of the sample: Fe content: 80 wt%, rigid matrix, x = 2 mm, maximum magnetic field $H_{max}=73$ kA/m.

**Figure 8:**
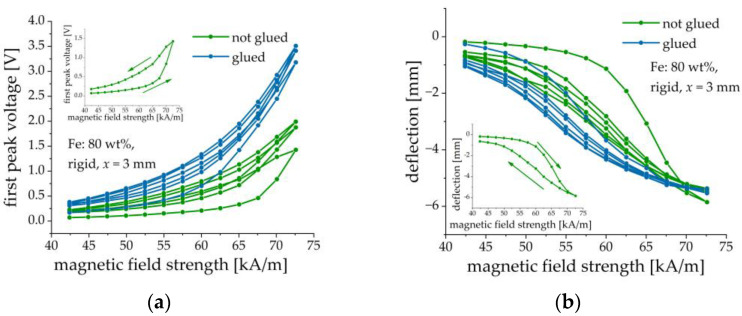
Comparison between composite structures with and without added silicone glue layer. (**a**) Dependence of the first peak voltage amplitude on the amplitude of magnetic field pulses. (**b**) Dependence of the cantilever’s deflection on the amplitude of magnetic field pulses. Parameters of the sample: Fe content: 80 wt%, rigid matrix, x = 3 mm. The insets illustrate the direction of field change for the first loop.

Makarova et al. [30] used either the inherent adhesion of the cured MAE layer to the PE substrate or cured the MAE layers on the PE substrate. To improve the adhesion between the MAE layer and PE element, we introduced a silicone glue layer between them. We verified that this silicone glue was non-magnetic. Without the silicone glue, the MAE layer may detach from the PE element after several loading cycles or longer exposure to a magnetic field, as it is illustrated in Figure 7. It was also found that the presence of an adhesive layer improves the performance characteristics of composite cantilevers, i.e., the deflection of the cantilevers with glued MAE layers is larger and the resulting output voltage is higher in comparison with the cantilevers, where the intermediate adhesive layer was absent. Figure 8 presents an example of such a comparison. In this and the following Figures, three consecutive magnetic-field loading cycles are shown. In one cycle, the amplitude of the magnetic field pulse was first increased from zero to the maximum value in 2.5 kA/m steps and then decreased from the maximum value to zero in the same steps. The field pulse length and the pause between the pulses were both equal to 2 s. If deflection is negative, this means that the cantilever is deflected down. If deflection is positive, the cantilever is deflected up. It is seen that the presence of a silicone glue reduces the hysteresis behavior of both the output voltage and the cantilever’s deflection in dependence on the applied magnetic field. We attributed this enlarged hysteresis to the creep of the MAE material at the MAE/PEP interface due to losing adhesion. In the following, the results refer to the MAE layers glued on the PE substrates.

### 3.3. Direction of Bending

One end of the cantilever was mechanically fixed in a clamp. This clamp was normally oriented horizontally. The magnetic field was applied in the vertical direction. We were not able to apply the magnetic field in the horizontal direction and to position the cantilever in the vertical plane due to the size and the weight of an electromagnet.

Under the effect of its own weight, the cantilever (in particular, the MAE layer) bent down a little in the absence of a magnetic field as can be seen in Figure 4. As explained in Ref. [26], the magnetization of a soft-magnetic beam upon its bending in a magnetic field, which is nominally perpendicular to the initial beam plane, is not co-directional with the magnetic field. If the magnetization is not collinear with the magnetic field, then a torque from the magnetic field acts on that beam. The same effect was observed in our case. We also verified that the bending effect of a soft-magnetic beam can be also observed in conventional materials without a restructuring of the filler. A thin strip from the nickel foil (EsportsMJJ 99.96% Pure Nickel Metal Foil, thickness = 0.1 mm) also bent in vertical magnetic fields, although higher magnetic fields were required to achieve the same bending magnitude in comparison to the MAE-PEP cantilevers. We concluded that this is the flexibility of the soft-magnetic layer which is required to realize bending, while the presence of filler restructuring seems to be not necessary.

**Figure 9:**
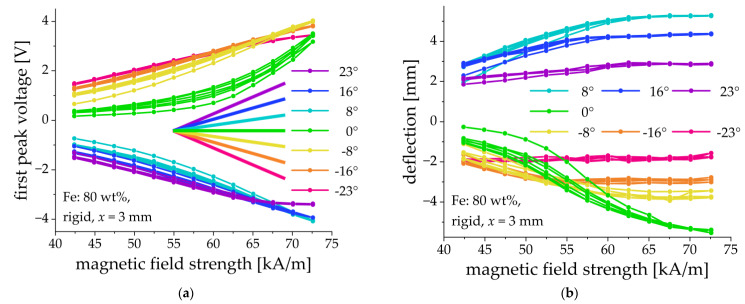
Field dependences of the first peak voltage amplitude (**a**) and the cantilever’s deflection (**b**) for different tilting angles of the clamp. Sample parameters: Fe content: 80 wt%, rigid matrix, x = 3 mm.

By changing the tilting angle of the cantilever’s clamp, it was possible to influence the direction of deflection as is shown in Figure 9. For example, a cantilever that was clamped horizontally (clamping angle α = 0°) deflected down (Figure 9a) and the peak voltage of the first output pulse had a positive sign (Figure 9b). A cantilever clamped at an angle of 8° up, deflected up (Figure 9a) and the peak voltage of the first output pulse was negative (Figure 9b). We concluded that the initial tilt of the clamp allows one to affect the preferential direction of bending. It was also observed that the hysteresis behavior of both the amplitude of the first voltage peak and the cantilever’s deflection was reduced when the fixation point was tilted. The majority of the fabricated cantilevers bent down when they were clamped horizontally, as expected. However, two fabricated cantilevers (70% and 75%, rigid matrix, x = 1 mm; see Section 3.4 below) bent up when a magnetic field was applied. An inspection of the PE substrates showed that some of them were not entirely planar, but they were slightly concave upward so that the upward bending appeared to be preferential. However, the behavior of these specific samples was not different from the rest of the fabricated cantilever, except for the direction of bending and the sign of output pulses.

**Figure 10:**
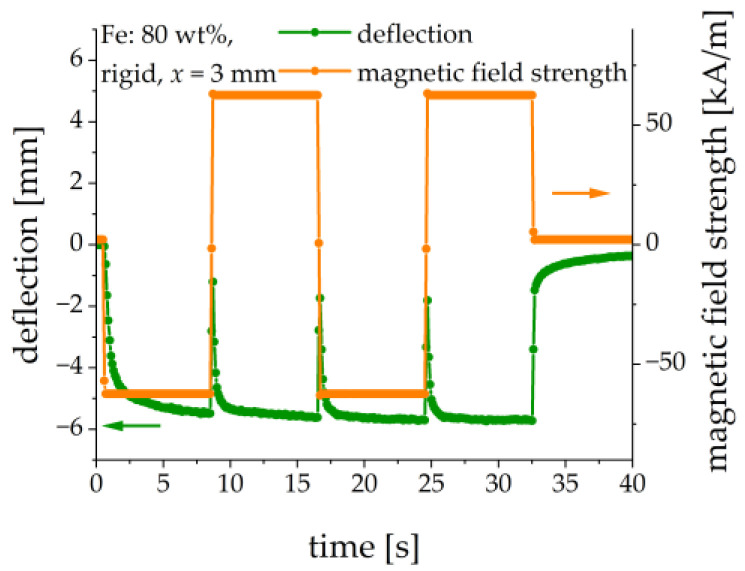
Comparison of the deflection and the direction of an applied square-wave magnetic field. Sample parameters: Fe content: 80 wt%, rigid matrix, x = 3 mm.

In [26], a beam from an MAE filled with nickel particles was used; the direction of the beam’s deflection changed with the change in the polarity of the applied magnetic field. This was explained by the presence of residual magnetization. In our control experiment, we clamped an MAE-PEP cantilever horizontally (α = 0°) and the polarity of the applied magnetic field was changed in a square-wave fashion, see Figure 10. It is seen that the deflection direction did not change, i.e., the MAE material seemed to behave as the particles would be ideally soft magnetic [26]. This result agreed with the measurements of magnetic properties. There was no significant remanent magnetization, which was able to change the direction of bending against the gravity force. Another difference of our polarity change experiment in comparison with that of [26] is the much higher ramp rate of the magnetic field. An MAE-PEP cantilever had a relatively large magneto-mechanical time constant with respect to the magnetic-field step excitation (see Section 3.6 below). Under presented experimental conditions the behavior of the MAE material with regard to the direction of bending seems to be reasonably considered as that of an ideally soft-magnetic material.

In higher magnetic fields, the deflection of a cantilever may become so large, that it will touch the bottom or top of the holder. This naturally limits the magnitude of the output voltage. This effect can appear as the “saturation” of the peak voltage, as it can be seen for the 23° and −23° curves in Figure 9. In what follows, the MAE cantilevers were clamped horizontally (α = 0°).

### 3.4. Comparison of Soft and Rigid Matrices

**Figure 11:**
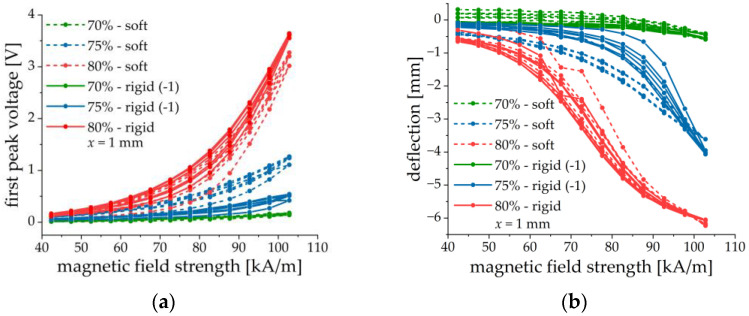
Field dependences of the first peak voltage amplitude (**a**) and the cantilever’s deflection (**b**) for two different matrices and three different iron contents. Parameters of MAE materials correspond to those of Table 1, x = 1 mm.

The effect of matrix stiffness was investigated on samples with 70, 75 and 80 wt% of CIP and a thickness x of the MAE layer of 1 mm. The results are summarized in Figure 11. As described above the samples denoted with (−1) defected upward. For comparison, the first peak voltage and the deflection were multiplied with −1.

No obvious dependence on the MAE stiffness is observed. Both the first peak voltage amplitude and the cantilever deflection have similar values for structures with different matrices but otherwise the same parameters. The ME and mechanical responses seem to be primarily determined by the iron content. This can be explained by the much higher elastic modulus of the PE substrate than of the MAE material (at least three orders of magnitude), even in a magnetic field. Therefore, the presence of MAE materials with slightly different elastic moduli (with respect to the elastic modulus of the PEP) may not significantly influence the mechanical behavior of a composite cantilever. However, it is clearly seen from Figure 11 that the peak voltage and the cantilever’s deflection tend to increase with the increasing concentration of iron particles. We speculate some differences in the voltage response of MAE-PEP cantilevers with different matrices but otherwise, identical parameters are due to some variations of the properties of PE substrates and fabrication uncertainties.

### 3.5. Effect of MAE Thickness

**Figure 12:**
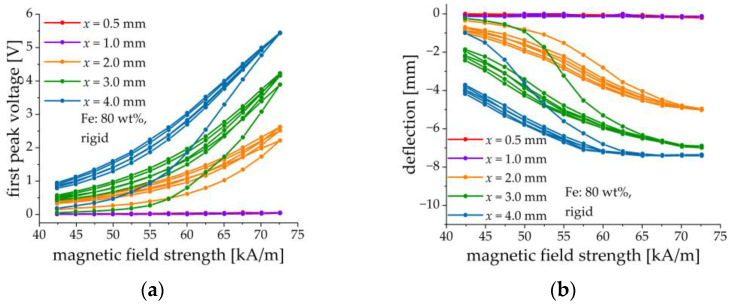
Field dependences of the first peak voltage amplitude (**a**) and the cantilever’s deflection (**b**) for different thicknesses of the MAE layer (80 wt% of Fe, rigid matrix).

The effect of MAE thickness is shown in Figure 12 on samples comprising MAE layers with an iron content of 80 wt% and a rigid matrix. Five different thicknesses were implemented: 0.5, 1, 2, 3, and 4 mm. Results are given in Figure 12. It is observed that both the deflection and the voltage response increase with the increasing thickness of the MAE layer. We believe that this is primarily due to the higher magnetic moment of the thicker samples and secondly because of the smaller demagnetizing factor of the thicker slab perpendicular to its surface. It can be also seen that the initial (magnetic field increase) curve significantly differs from the subsequent cycles. It can be expected that during the first cycle major restructuring of the filler takes place, whereas further changes are minor [46].

### 3.6. Transient Behavior

The deflection of a composite cantilever did not follow the magnetic field instantaneously. Such a retarded behavior to magnetic-field step-like excitations was observed before for rheological [47] and dielectric properties of MAEs [46]. The explanation was that the particles can move and rearrange themselves within a compliant elastomeric matrix [48]. Moreover, since MAE is a viscoelastic material, it is natural to expect transient behavior upon its loading.

**Figure 13:**
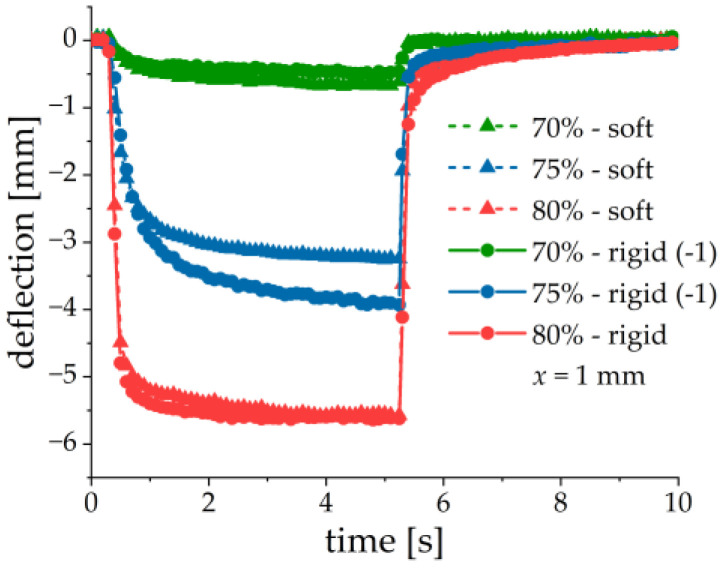
Transient response of cantilever’s deflection to the magnetic field pulse with the amplitude H=103 kA/m. MAE materials correspond to those of Table 1, x = 1 mm.

Figure 13 presents the measured deflection upon application of a magnetic field pulse with a length of 5 s. The time dependence upon both magnetic-field loading and unloading was very well described by a single exponential function. Table 2 shows the determined time constants $\tau_{on/off}$ and the coefficient of determination $R^{2}$. When the magnetic field is switched on, the time constant decreases with the increasing iron content. This can be explained by spatial limitations for particle rearrangements in higher filled samples. For samples with 70 wt% of iron, the time constant $\tau_{on}$ is about 30% larger in the sample with a softer matrix, which can be explained by the higher mobility of filler particles in a softer matrix. The difference between time constants $\tau_{on}$ in samples with 75 and 80 wt% of iron is not so clearly pronounced, they are more similar $\tau_{on}$ is about 20% larger in 75% (rigid sample) than in 75% (soft sample). For 80% (rigid and soft samples), $\tau_{on}$ is the same within the accuracy of measurements. When the magnetic field is switched off, the time constants $\tau_{off}$ are much smaller and similar, they have the order of magnitude of the time scale of the magnetic field ramp: $103\;{\mathrm{kAm}}^{-1}/770\;{\mathrm{kAm}}^{-1}s^{-1}\approx 0.13\;s$. When the magnetic field is turned on, the elastic force from the matrix resists the rearrangement of filler particles and it accelerates the relaxation process when the magnetic field is turned off, by forcing the particles towards their initial positions.

## 4. Discussion

The bending mechanism of the composite MAE–PEP cantilever is described in Ref. [26]. If a soft-magnetic planar MAE cantilever would be directed perpendicular to the uniform magnetic field, it would not be bent. A slight inclination of the normal to the MAE plane with respect to the external magnetic field leads to the torque acting on the MAE layer by the magnetic forces, because the magnetization of the MAE layer and the external magnetic field are not collinear. This torque tries to align the MAE slab parallel to the external magnetic field, in order to minimize its demagnetizing factor. Because the MAE layer is mechanically coupled to a flexible PE substrate, the entire structure is bent. The composite structure is made from flexible materials, therefore, the resulting deformation of the PE material is high, which leads to high output voltages. The maximum amplitude of the first voltage peak in the present work is 5.5 V, which is about 8.5-fold larger than the maximum voltage reported in [30]. It is observed on the sample with 80 wt% of Fe, rigid matrix and a thickness x of 4 mm sample in a magnetic field H=73 kA/m. The appearance of the electrical voltage between the electrodes of the PE material as a result of an applied magnetic field can be considered as the manifestation of the direct ME effect in an MAE-PEP structure. It is the mechanical strain that mediates the ME coupling in our case like in traditional (layered) ME heterostructures. In traditional layered heterostructures, the application of an external magnetic field perpendicular to the sample’s plane is not efficient, because of the large demagnetizing factor. In our case, the magnetostrictive material (MAE) is extremely soft (Young’s modulus ~10–100 kPa), while the PEP is flexible. This leads to large deformations and large output voltages.

The main factor affecting the amplitude of the output voltage and the cantilever’s deflection seems to be the total quantity of iron in the MAE layer, because of the total magnetic moment induced in it and the resulting torque on the composite structure, see Figure 11 and Figure 12. A larger amount of iron filler leads to a higher magneto-mechanical response of the cantilever and to more efficient ME coupling. MAE layers with soft and rigid matrices give similar results because the difference in their stiffness can be neglected in comparison with the stiffness of the PEP.

The usage of an additional adhesive layer between the MAE and PEP provides improvements to the composite structures, not only as far as the structure mechanical stability (avoiding delamination) is concerned, but also increases the output voltage and reduces the hysteresis.

The direction of deflection can be efficiently controlled by the initial tilt of the clamping point in order to compensate for the effect of gravity. However, if the MAE-PEP cantilever is fixed horizontally, such a position is unstable in a magnetic field and depends on the geometrical details, such as the deviation of the PE substrate from the planarity.

**Figure 14:**
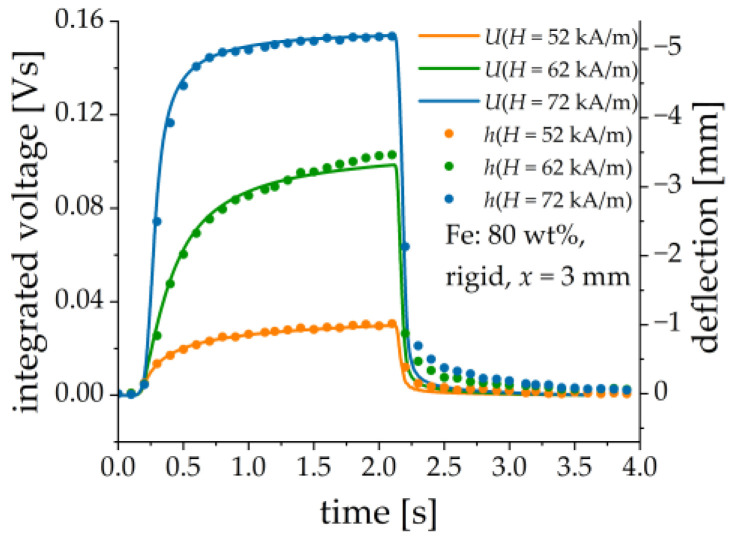
Comparison between numerically integrated output voltage U (continuous lines) and the cantilever’s deflection h (dots) for three different magnetic field pulses. Sample parameters: Fe content: 80 wt%, rigid matrix, x = 3 mm.

It is found that the output voltage can be reasonably described as proportional to the first-time derivative of the cantilever’s deflection, see Figure 14. This can be explained by a simple model. Assume that the generated charge is linearly proportional to the deflection *h*: Q=kh, where k is some constant. The simplified equivalent circuit of the PE transducer is a current source with the source current $I_{q}=kdh/dt$ and a capacitor $C_{eq}$ and a resistor $R_{eq}$ connected in parallel. The values of concentrated elements $C_{eq}$ and $R_{eq}$ are determined by the PE element and the measurement circuit, e.g., the connecting cable. The corresponding time constant of the equivalent circuit is $\tau_{eq}=R_{eq}C_{eq}$. The time dependence of the voltage U is described by the following inhomogeneous differential equation of the first order:

(1) $$U\left(t\right)+\tau_{eq}\frac{dU}{dt}=kR_{eq}\frac{dh}{dt}.$$

If U(0)=0, the solution to Equation (1) can be written as:

(2) U(t)=e−tτeq[kReqTeq∫0tet′τeq(dhdt′)dt′].

The voltage U(t) is proportional to dh/dt, if the time constant $\tau_{eq}$ is small enough, that can be also seen from Equation (1) when |U|≫|τeqdUdt|.

**Figure 15:**
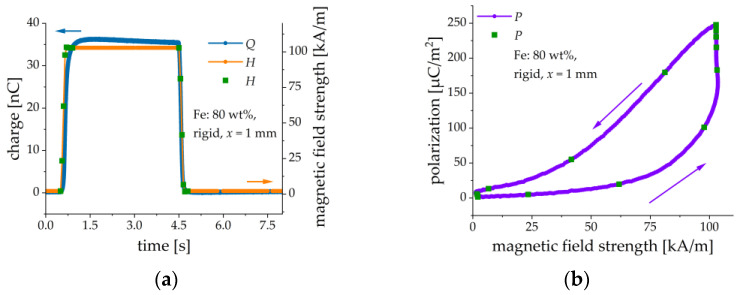
(**a**) Time dependences of the external magnetic field H and the magnetically induced charge Q. (**b**) Calculated dependence of the electrical polarization P on the magnetic field strength H. Green dots on the H-field time dependence are given to illustrate the corresponding magnetic fields in both diagrams.

Let us estimate the magnetically induced ME coupling coefficient:

(3) $$\alpha^{H}=\frac{\partial P}{\partial H},$$

for a particular MAE-PEP heterostructure with 80 wt% of Fe, rigid matrix and x=1 mm. This sample is selected among others for demonstration because it has a high ME response and the largest working range of H. In (3) the external magnetic field H is directed vertically and P is the resulting electrical polarization. From the material equation $D=\varepsilon_{0}\varepsilon_{r}E=\varepsilon_{0}E+P,$ we obtain that $P=D{\left(1-\varepsilon_{r}^{-1}\right)}^{-1}=\sigma {\left(1-\varepsilon_{r}^{-1}\right)}^{-1}$, where E is the induced electric field, D is the electric displacement, $\varepsilon_{0}$ is the vacuum permittivity, $\varepsilon_{r}$ is the relative permittivity of PE material, and σ is the surface charge density on the electrodes of PE material. The surface charge density is measured by integration of the output current $i_{out}$ by a charge amplifier, divided by the area A of the PE layer from the datasheet: σ=Q/A, where $Q=\int^{}i_{out}dt$ is the generated electric charge. The material constant $\varepsilon_{r}$ is assumed to have the value $\varepsilon_{r}\approx 15$ [49]. Figure 15 presents the results of the measurements. A slow decline of the induced electrical charge in the constant magnetic field is attributed to the non-perfect characteristics (leakage) of the charge amplifier. We did not use this part of the transient behavior of the charge Q(t) for the calculation of the ME coupling coefficient. In Figure 15a the measuring rate for H is 20 pts/s and 1000 pts/s for the charge Q. Therefore, a cubic spline algorithm was used to interpolate between measured points of H. As described above, the deflection of the MAE-PEP cantilever is delayed with respect to the applied magnetic field. Therefore, a clear hysteresis behavior of the electrical polarization with respect to the applied magnetic field is observed in Figure 15b.

**Figure 16:**
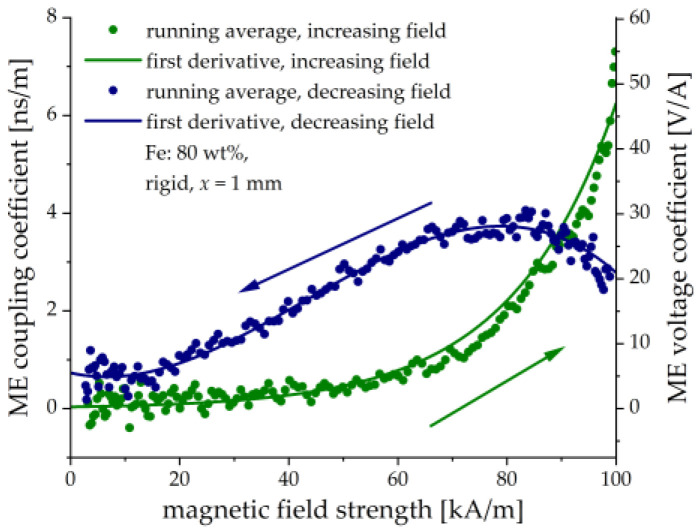
Calculated field dependences of the ME coupling coefficient $\alpha^{H}$ and the ME voltage coupling coefficient $\alpha_{V}^{H}$.

To determine the ME coupling coefficient $\alpha^{H}$, the derivatives in (1) have to be calculated numerically from experimental data with some noise, which generally results in the scattering of calculated values. The dots in Figure 16 represent the numerical first derivatives between the neighboring points and subsequent usage of the running average over five points. The continuous lines show the first derivatives of the fitted analytical functions. For the increasing magnetic field, the following empirical dependence of the polarization P on the magnetic field H is used: $P\left(H\right)=\left(c+bH\right)e^{aH}$. For the decreasing magnetic field, a polynomial dependence is used: $P\left(H\right)=a_{0}+a_{1}H+a_{2}H^{2}+a_{3}H^{3}+a_{4}H^{4}$. The derivatives are then calculated analytically. We also recalculated the ME coupling coefficient $\alpha^{H}$ into the magnetically induced voltage ME coefficient $\alpha_{V}^{H}=\alpha^{H}{\left(\varepsilon_{0}\varepsilon_{r}\right)}^{-1}$ [34,50].

It is seen in Figure 16 that the field behavior of the ME coupling coefficient differs strongly for the ascending and descending magnetic fields. There is a monotonic increase in $\alpha^{H}$ with the increasing magnetic field and a local maximum in the decreasing magnetic field. We are not aware of any similar behavior observed in traditional (metal/ceramics) layered heterostructures. Of course, the observed hysteresis is related to the delayed response between the applied magnetic field and the induced electrical polarization. It is seen from Figure 15, that the electric charge continues to grow even when that magnetic field reached the steady-state. This is because the cantilever’s deflection still takes place. Formally, such behavior would result in an infinite ME coupling coefficient, which is non-physical and, hence not shown in Figure 16. The reason is that Equation (3) implies the instantaneous ME response, which is not the case in our paper. We intended to investigate the field dependence of the ME coupling coefficient and its transient behavior in more detail in the following works. The maximum value of the ME voltage coefficient is about 50 V/A (≈40 V/(cm Oe) in cgs units). It compares well with conventional layered heterostructures, in particular taking into account that it was obtained in a quasi-static magnetic field on the non-optimized structure. The ME coefficient can be increased by using thicker MAE layers, optimized PE substrates with large PE coefficients and flexibility and, likely, by operation at the resonant frequency of the resulting heterostructure. Further research is required on whether it is possible to significantly reduce the time constant for the ME response and to minimize hysteresis.

## 5. Conclusions

Detailed systematic investigations of the magnetically induced bending and the generated voltage are performed on the new type of ME heterostructures in quasi-static pulsed magnetic fields. In comparison with a previously published work [30], ME structures are exposed to uniform magnetic fields. The magnitude of the voltage response increases with the increasing total amount of soft magnetic filler in the MAE layer. Application of a homogeneous magnetic field to our MAE-PEP heterostructures results in an 8.5 higher-generated voltage than previously reported. We attribute this enhancement of the response to the improved fabrication procedure using an adhesive layer between the MAE and the PEP. The general relationship between the cantilever’s deflection and the generated voltage is established. The application of a uniform magnetic field allowed us to estimate the ME coupling coefficient from the measurement of the generated electric charge in these types of layered composites for the first time. Under quasi-static conditions, the magnetically induced ME voltage coefficient can be as high as ≈50 V/A in the measurement range of 100 kA/m. Obtained results confirm the previously put proposal in [30] that these novel structures are promising for sensors and energy harvesting devices.

Further research is required to understand the unconventional field behavior of the ME coupling coefficient in MAE-PEP heterostructures and to find the conditions for eliminating the observed hysteresis of the generated voltage in the applied magnetic field while keeping the high responsivity to magnetic fields. It would be interesting to miniaturize investigated magneto-sensitive elements, e.g., by microsystem technology, and to characterize them by the method of harmonic field modulation. We believe that the employment of mechanically soft ME composite structures may lead to the change in the design paradigm for highly sensitive ME sensors and energy harvesting devices. Furthermore, the electrical voltage generated in such structures can be utilized for self-sensing in magnetically controlled actuators or grippers as was proposed in Ref. [27].

## Figures and Tables

**Table 1:** Shear storage modulus $G_{0}^{\prime }$ of the matrices and the synthesized MAE samples.

Fe Mass Fraction [wt%]	Fe Volume Fraction [vol%]	$G_{0}^{\prime }$ [kPa]—“soft”	$G_{0}^{\prime }$ [kPa]—“rigid”
0	0	0.5	10.1
70	22	3.9	33.1
75	27	5.2	34.5
80	33	11.6	50.4

**Table 2:** Time constants and determination coefficients for switching magnetic fields on and off.

	$\tau_{on}\;[s]$	$R^{2}$	$\tau_{off}\;[s]$	$R^{2}$
**70%—soft**	0.73 ± 0.05	0.96	0.23 ± 0.02	0.88
**75%—soft**	0.41 ± 0.02	0.98	0.11 ± 0.01	0.96
**80%—soft**	0.24 ± 0.02	0.94	0.20 ± 0.01	0.96
**70%—rigid**	0.55 ± 0.05	0.93	0.10 ± 0.01	0.94
**75%—rigid**	0.50 ± 0.02	0.98	0.09 ± 0.01	0.97
**80%—rigid**	0.22 ± 0.02	0.94	0.14 ± 0.01	0.97

## Data Availability

The data that support the findings of this study are available from the corresponding author, G.G., upon reasonable request.
